# Physical Activity With Tailored mHealth Support for Individuals With Intellectual Disabilities: Protocol for a Randomized Controlled Trial

**DOI:** 10.2196/19213

**Published:** 2020-06-29

**Authors:** Henriette Michalsen, Silje Camilla Wangberg, Gunnar Hartvigsen, Letizia Jaccheri, Miroslav Muzny, André Henriksen, Monica Isabel Olsen, Gyrd Thrane, Reidun Birgitta Jahnsen, Gunn Pettersen, Cathrine Arntzen, Audny Anke

**Affiliations:** 1 Department of Rehabilitation University Hospital of North Norway Tromsø Norway; 2 Department of Clinical Medicine University of Tromsø – The Arctic University of Norway Tromsø Norway; 3 Department of Health and Care Sciences University of Tromsø – The Arctic University of Norway Narvik Norway; 4 Department of Computer Science University of Tromsø – The Artic University of Norway Tromsø Norway; 5 Department of Computer Science The Norwegian University of Science and Technology Trondheim Norway; 6 Department of Community Medicine University of Tromsø – The Arctic University of Norway Tromsø Norway; 7 Department of Health and Care Sciences University of Tromsø – The Arctic University of Norway Tromsø Norway; 8 Department of Neurosciences for Children Oslo University Hospital Oslo Norway; 9 Research Centre for Habilitation and Rehabilitation Models and Services University of Oslo Oslo Norway

**Keywords:** intellectual disability, physical activity, technology, mHealth, mobile phone, goal attainment, social support, self-efficacy

## Abstract

**Background:**

Individuals with intellectual disabilities (IDs) have lower levels of physical activity (PA) and greater barriers for participation in fitness activities compared with members of the general population. As increased PA has positive effects on cardiovascular and psychosocial health, it is exceedingly important to identify effective interventions for use in everyday settings. Mobile health (mHealth) methods such as motion sensor games (exergames) and smartphone reminders for PA have been explored and found to be promising in individuals with IDs.

**Objective:**

The purpose of this study is to examine the effectiveness of an individually tailored PA program with motivational mHealth support on daily levels of PA in youth and adults with IDs.

**Methods:**

The trial uses a randomized controlled design comprising 30 intervention participants and 30 control group participants, aged 16 to 60 years, with sedentary lifestyles or low PA levels. While the controls will receive standard care, the intervention aims to increase the level of PA, measured as steps per day, as the primary outcome. Secondary outcome variables are body mass index, blood pressure, physical performance, social support for PA, self-efficacy in a PA setting, behavior problems, and goal attainment. The intervention involves the delivery of tailored mHealth support, using smartphones or tablets to create structure with focus on the communicative abilities of individual participants. Rewards and feedback are provided in order to motivate individuals to increase participation in PA. Participants in the intervention group, their close relatives, and care staff will be invited to participate in a preintervention goal-setting meeting, where goal attainment scaling will be used to select the participants’ PA goals for the intervention period. All participants will be assessed at baseline, at 3 months, and at 6 months.

**Results:**

Enrollment was planned to start in April 2020 but will be delayed due to the pandemic situation. The main contribution of this paper is a detailed plan to run our study, which will produce new knowledge about tailored mHealth to support PA in individuals with intellectual disabilities.

**Conclusions:**

We expect the new intervention to perform better than standard care in terms of improved PA, improved self-efficacy, and social support for activities. Technology offers new opportunities to promote healthy behaviors. The results of the study will determine the effectiveness and sustainability of a tailored mHealth support intervention to increase PA in youth and adults with IDs.

**Trial Registration:**

ClinicalTrials.gov NCT04079439; https://clinicaltrials.gov/ct2/show/NCT04079439

**International Registered Report Identifier (IRRID):**

PRR1-10.2196/19213

## Introduction

The prevalence of intellectual disabilities (IDs) is estimated to be 1% of the world’s population [1,2]. Compared with the general population, individuals with IDs have worse health and lower levels of activity [3-5], and they have greater barriers for participating in fitness activities [6] and accessing health care services [7,8].

### Physical Activity Guidelines

The World Health Organization (WHO) guidelines for physical activity (PA) state that typical adults should spend a minimum of 150 minutes per week engaged in moderate-intensity PA or 75 minutes engaged in vigorous-intensity PA [9]. One systematic review found that only 9% of individuals with IDs met the WHO´s minimum PA guidelines [4]. Norwegian guidelines are in line with the international guidelines and recommend 150 minutes of moderate- to vigorous-intensity PA per week for adults [10] and 60 minutes per day for children and youths [11]. As high PA is a determinant of health and increased activity has positive effects on cardiovascular and psychosocial health, identifying effective interventions for use in everyday settings is exceedingly important.

### Physical Activity Interventions

Some well-designed studies have not been able to demonstrate improved levels of PA in intervention groups of individuals with IDs after the intervention period has ended. One theory-based randomized controlled study of adults with all types of IDs did not find any significant increases in levels of PA (steps per day) using a walking program [12]. Furthermore, the results of a cluster-randomized study of older adults in the Netherlands showed marginal effects and substantial missing data, despite being well prepared with a published protocol and using day-activity centers for the intervention [13]. Past controlled studies on the effects of PA interventions on individuals with IDs have primarily included adults with mild to moderate IDs, and effect sizes have been small [5,14]. Some studies have reported improved effects on physical fitness indicators such as balance and muscle strength [15], psychological well-being [16], perception of social competence [17], and work routines [14] after increasing levels of PA. One recent study included individuals with severe or profound IDs in a technology-aided program for promotion of PA and found positive results in energy expenditure and independent engagement in light to moderate PA [18], but with a small number of participants. Findings from previous studies indicate that a more flexible approach [19], greater use of theory in intervention design, stronger research design (as there are few randomized controlled studies), and better translation of interventions to community-based settings [20] are needed. Future studies could also have an ecological approach, where the interplay between individual, interpersonal, and environmental factors are considered [1,21]. Motivational issues have been challenging, particularly for approaches that aim for sustainable effects [17], and should be considered when designing future PA interventions.

### Mobile Health Interventions

Mobile health (mHealth) provides a wide range of possibilities for monitoring and motivating individuals in the self-management of chronic illnesses [22-24]. Motion sensor games (exergames) have been explored and have been found to be promising in individuals with IDs [25]. For these solutions to move out of the lab and into actual use, they need to first meet users’ needs [26]. The mobile platform is ubiquitous, and the touch interface has proven to have a low level of cognitive demand and could be used to improve adherence to PA [27]. At present, few mobile apps have incorporated games strategies, such as goal setting or rewards, to increase PA in individuals with various disabilities [28]. To our knowledge, there has been only 1 preliminary report (letter) of a randomized controlled trial using smartphone support to increase motivation for PA in youth and adults with IDs [29].

Methods that could facilitate the development of individualized mHealth support solutions include tailoring, individual goal setting [30], and adjusting communication to meet the specific needs of individuals with IDs [31]. Studies on motivation for PA in the IDs population have shown that predictability with routine and familiarity, communication of purpose, and enjoyable and social activities promote motivation and participation [21,32]. Family and care staff involvement will be central, and we expect the study’s implementation in a natural setting to enhance the effect [33]. We also expect the systematic use of mHealth with rewards and gamification to be beneficial. In Norway, many individuals with IDs have a smartphone or a tablet that they can use for tailored PA interventions. However, this use has not been tested previously. We expect a motivational app for smartphones and tablets to promote adherence to PA in individuals with IDs. According to the *World Report on Disability*, health promotion efforts targeting this population can improve lifestyle behaviors [34]. The report states that these individuals have the right to be included in all PA programs. Thereby, the present study aims to examine the effectiveness of an individually tailored PA program with motivational mHealth support on everyday levels of PA in youth and adults with IDs, targeting individual, contextual, and interactional factors of PA participation [21]. In addition to higher levels of PA, we expect improvements in psychological health, self-efficacy in activities [32], and social support for physical activity participation [35].

## Methods

### Design

The current study has a randomized controlled clinical design with assessments at baseline, 3 months, and 6 months. Participants will receive either the tailored mHealth-supported PA program or standard care with access to the mHealth support system once the trial is completed. Family and care staff will be involved in the program for support and follow-up.

### Participants

A total of 60 participants will be recruited into the trial through health care and other related organizations in the municipality of Tromsø in northern Norway. Information about the study will be delivered at meeting places, such as day and activity centers. If the number of included participants is insufficient, more municipalities in Northern Norway will be included, or a multicenter approach will be considered.

### Inclusion and Exclusion Criteria

Individuals will be included if they have a sedentary lifestyle [1] or a low level of PA, determined with the question, “In how much of your leisure time have you been physically active in the last year?” [8]. The question has 4 response categories and has been used in population-based studies of the general population [36] and in European health indicator studies of individuals with IDs [8]. Individuals with a sedentary lifestyle (eg, primarily engaged in reading, watching television, or other mainly sedentary activities) and a low level of PA (eg, engaged in walking or other light PA for less than 4 hours a week) will be included in the study. Other inclusion criteria will be as follows: (1) diagnosis of ID (mild, moderate, severe, or profound), (2) aged between 16 and 60 years, (3) ability to participate in the intervention, (4) ability to walk with or without support, and (5) able to provide written informed consent or written informed consent can be obtained from a representative. Prior to enrollment, all participants will be screened for readiness, and, if necessary, medical clearance will be obtained. The Physical Activity Readiness Questionnaire [37] will be used for this purpose. Exclusion criteria will be as follows: (1) medical contraindications for participation in programs with increased exercise as advised by the primary care or ID specialist physician, (2) high level of physical activity, and (3) inability to provide written informed consent and written informed consent cannot be obtained from a representative.

### Ethics

This study has been approved by the Regional Committee for Medical and Health Research Ethics in northern Norway (number 2016/1770). The protocol has been registered at ClinicalTrials.gov under the identifier NCT04079439. The project adheres to the Consolidated Standards of Reporting Trials guidelines. Written informed consent will be obtained from each participant, their legal representative, or both prior to inclusion in the study and baseline assessment. If the participant has impaired capability to consent, consent will be sought from the nearest relative or guardian as well as from the individual with IDs, or as representative consent. Ethical issues will be continuously considered. Any adverse events will be recorded.

### Randomization

Participants will be randomized with a computer program to either the PA intervention with mHealth support group or the standard care control group.

#### Intervention Group

**Figure 1:**
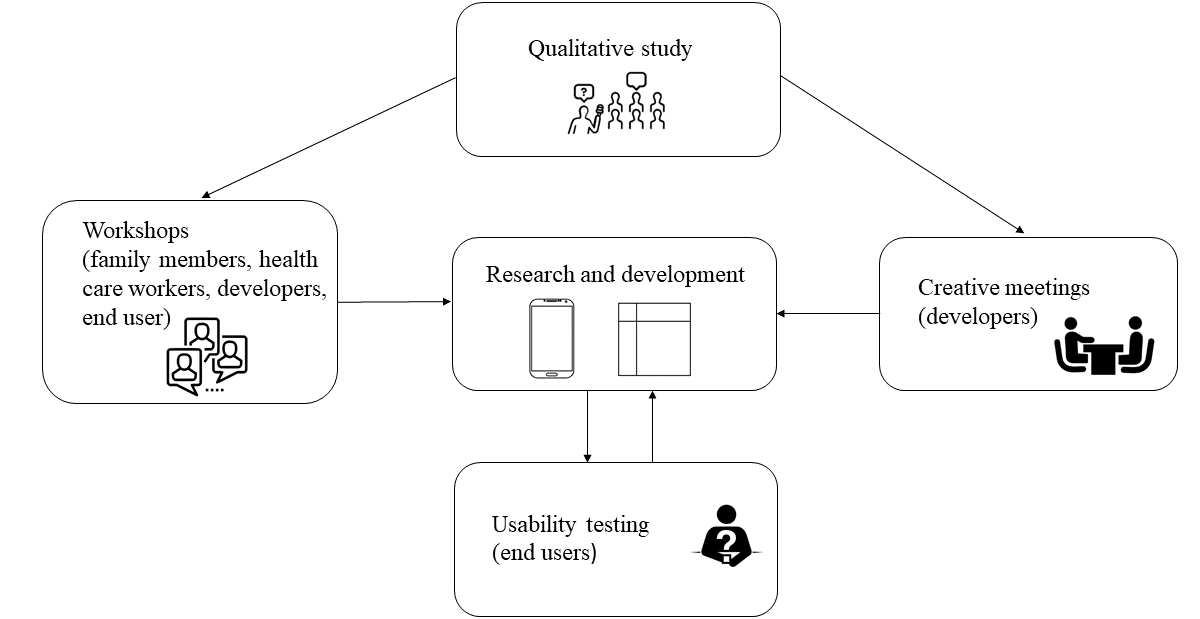
Development process of the electronic health support component of the study.

This randomized controlled trial is part of our project to develop a tailored mHealth support system that promotes PA in individuals with IDs [38]. In previous parts of the project, we conducted a qualitative study on motivation for participation in PA for individuals with IDs [21], held workshops and collaborated with mHealth developers, and performed usability tests. This process is illustrated in Figure 1.

**Figure 2:**
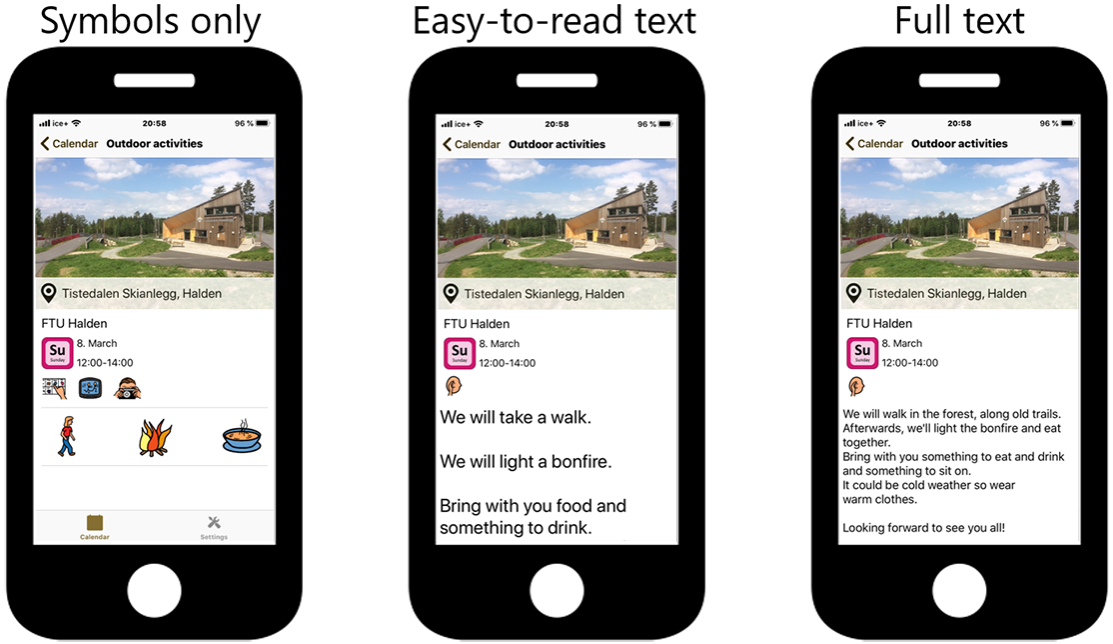
Interface options of the Actiplan app: symbols only, easy-to-read text, or plain text. The app also has read-aloud capabilities.

Findings from the qualitative study, discussions in workshops, and creative meetings among developers and researchers showed that many individuals with IDs experience difficulties participating in PA because of individual, interactional, and contextual factors. Some of these factors include individual difficulties in initiating activities; preferences for fun and social activities; and lack of social support, availability of activities, resources and preparation, predictability, and collaboration in activities. After examination of these findings, a prototype of an app was created and presented in one of the workshops. The feedback was promising, and development of the mHealth support system continued. The main emphasis in the app is individually chosen activities, tailored communication, predictability, use of rewards, and involvement of support persons. Activities will be chosen during a goal attainment meeting (using goal attainment scaling) [39] with the individual with ID and a support person (usually a family member or health care provider). Goal attainment is widely used as an outcome measure within rehabilitation medicine [39] and has been used in studies with individuals with IDs [40]. The research group is familiar with the use of goalsetting processes in previous studies with individuals with IDs. The final intervention will consist of an advanced activity planner based on the platform for the app Active Leisure (Smart Cognition AS). Actiplan features and tracks daily physical activities. The app offers different interface options (symbols only, easy-to-read text, plain text, and read aloud). See Figure 2 for examples. The activity planner will mostly be used by the individual with ID and a support person (caregiver or health care provider). After completing an activity, a simple reward is available (eg, a smiling face or sharing a picture with other users of the app). The use of tailored communication [41] is available through personalization, including the use of the individual’s first name in the activity planner, individually chosen activities, adjusted communication (eg, symbols, easy-to-read text, or plain text), preparation and planning, and feedback. Rewards and positive feedback after activities have been performed are expected to increase motivation, and thereby lead to higher levels of PA.

In addition, 3 individual exercise apps have been developed as potential alternatives that can be added to Actiplan: (1) an exergame or game-inspired app that promotes outdoor PA; (2) an ergometer bike or outdoor bike placed inside the home, with a mounted screen showing a video or other visually interesting features; and (3) a game-inspired, avatar-based health platform for monitoring PA and motivating users to increase their PA levels [38].

To explore the participants’ expectations and experiences of the intervention and the mHealth support, a qualitative pilot study recruiting 10 of the first participants in the intervention group will be performed.

#### Control Group

Participants in the control group will be administered the assessments at baseline, 3 months, and 6 months, and otherwise continue with their standard everyday activities during the study period. They will be invited to use the mHealth support system at the end of the 6-month intervention period.

### Data Collection

**Figure 3:**
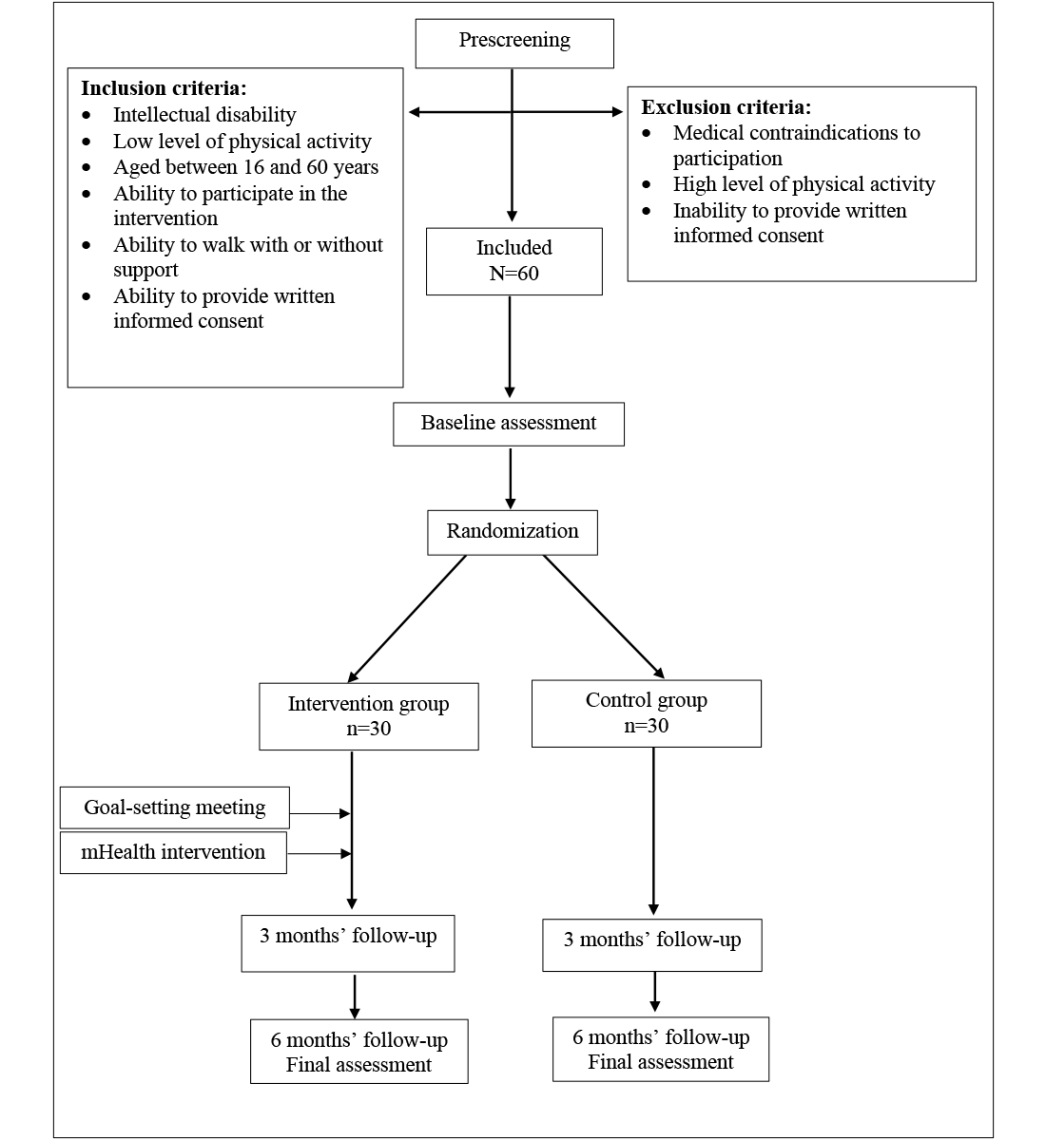
Consolidated Standards of Reporting Trials flow diagram of the study. mHealth: mobile health.

Data will be collected at baseline, 3 months, and 6 months, as seen in Figure 3. Baseline data will include baseline PA activity level and will be collected before randomization. Follow-up data will be collected regardless of the participant’s compliance with the study protocol. Participants and assessors will not be blinded. Background data on the participants will be collected at baseline and will include age, gender, educational level, marital status, living condition, employment status, educational status, job-related or day center activities, leisure time activities, smoking habits, level of ID (ie, mild, moderate, severe, or profound), genetic diagnosis, gross motor function classification [42,43], communication level [44], medical history/readiness for the PA intervention, and use of medication. In addition, we will ask questions about barriers for participation in physical activities.

### Outcome Measures

#### Primary Outcome

The primary outcome of this study will be objectively measured PA, as assessed by steps per day measured with a wrist-worn commercial accelerometer (Fitbit Versa; Fitbit Inc). The device will assess level of PA and sedentary time [45]. The watch will be covered (neutral screen) during baseline and follow-up assessments in both the intervention and control groups. Screen neutrality is achieved by running a custom app on the watch that disables all buttons and prevents screen feedback (except showing current time). This app cannot be disabled by the participant. Level of PA will be measured for 7 days at each assessment, with a minimum of 3 consecutive days of measurement because previous research has shown that 3 days of PA can predict the weekly level of PA [14,46]. Many of the commercial fitness trackers have been validated for use in research [47], including devices from Fitbit Inc [48,49].

#### Secondary Outcomes

Secondary outcome measures will include minutes of moderate PA per day, PA questionnaire, body mass index, blood pressure, physical performance, social support for physical activity, self-efficacy in a PA setting, behavior problems, and goal attainment. See Table 1 for a summary of all outcome measures.

**Table 1:** Summary of outcome measures.

Measurement	Type of outcome	Measure
Steps per day	Primary outcome	Fitness tracker
Minutes of moderate activity	Secondary outcome	Fitness tracker
Body mass index	Secondary outcome	kg/m^2^
Blood pressure	Secondary outcome	Blood pressure monitor
Physical performance	Secondary outcome	Short Physical Performance Battery [50]
Behavior problems	Secondary outcome	Aberrant Behavior Checklist–Community [51]
Social support for PA and self-efficacy in PA setting	Secondary outcome	The Self-Efficacy/Social Support for Activity for Persons With Intellectual Disability scale [35]
Goal attainment	Method, secondary outcome	Goal attainment scaling [39]
Satisfaction with life	Control for adverse effects	Satisfaction with life scale [52]

##### Physical Activity

The secondary PA outcome is the number of minutes of moderate PA per day, measured with the commercial accelerometer.

In addition, the International Physical Activity Questionnaire-Short Form, adapted to measure PA using proxy respondents, will be used [53]. The International Physical Activity Questionnaire-Short Form is a 7-item questionnaire that assesses PA in the past 7 days at 4 intensity levels: (1) vigorous-intensity activity, such as aerobics, (2) moderate-intensity activity, such as leisure cycling, (3) walking, and (4) sitting.

##### Body Mass Index and Blood Pressure

Body mass index will be calculated in kg/m^2^ [54]. Blood pressure will be measured using a blood pressure monitor (Welch Allyn Inc). Height will be measured in meters with a stadiometer (Seca GmbH), with the participant wearing no shoes. Weight will be measured in kilograms with an analog floor scale (Seca GmbH), with participants wearing no shoes or outdoor jackets/gear. Waist circumference will be measured 1 cm above the navel.

##### Physical Performance

The Short Physical Performance Battery (SPPB) will be used to assess physical performance. The SPPB is a screening test designed to evaluate physical performance and predict disability in older adult populations [50]. The SPPB is mainly a measure of lower-extremity function and consists of 3 subtests: static balance, gait speed, and lower limb strength. To measure static balance, the participant is asked to stand with feet in the side-by-side, semitandem, and tandem positions, for 10 seconds each, to his or her best ability. Gait speed is measured with a 4-m (13-ft) walk at the individual´s habitual pace. Lower limb strength is measured by having the participant rise from a chair with arms folded across his or her chest, to his or her best ability. Subtest scores range from 0 (inability perform the test) to 4 (highest level of performance). The SPPB has been validated [55], and reference values have been published [56]. The Norwegian version of the SPPB appears to have acceptable reliability as well as good internal consistency in an older population with and without dementia [57]. The SPPB has been used in people with mild and moderate IDs [58,59].

##### Behavior Problems

The Aberrant Behavior Checklist—Community (ABC-C) is a questionnaire designed to assess challenging behavior in children, youth, and adults with IDs [51]. The checklist consists of 58 items divided into 5 subscales: irritability, lethargy, stereotypy, hyperactivity, and inappropriate speech. It is a proxy measure requiring knowledge of the person with ID. Each item is scored on a scale of 0 to 3 (3 indicating the highest severity). The ABC-C is a widely used behavioral rating scale used among people with IDs. A Norwegian study [60] examined the construct validity of the Norwegian ABC in a clinical sample of children and youths in Norway and found satisfactory psychometric properties for the ABC, with the exception of the inappropriate speech factor. The Cronbach coefficients were adequate to excellent, with coefficients ranging from .76 to .95. The ABC subscales were moderately to highly correlated with one another (*r*=0.41-0.78, *P*<.001).

##### Social Support for Physical Activity and Self-Efficacy in a PA Setting

The Self-Efficacy/Social Support for Activity for Persons with Intellectual Disability scale [35] is a questionnaire consisting of 4 subscales. One subscale measures self-efficacy for overcoming barriers to leisure PA. The last 3 subscales measure social support for leisure activity from family members, care staff, and friends for individuals with IDs. The scale has been validated for self-reporting from individuals with mild to moderate IDs and for use by proxy respondents [35]. The questionnaire will be translated into the Norwegian language using standard guidelines [61].

##### Goal Attainment

Goal attainment scaling [39,62] will be used to identify self-management goals that participants would like to achieve. The questionnaire will be filled out by the researcher, with participants and proxy respondents present. Goal attainment scaling involves several steps. Goals are selected by each individual, and observable behavior that reflects a degree of goal attainment is defined [63]. The participant’s pretreatment or baseline levels are defined in terms of the goal. Five different goal attainment levels are used, ranging from “no change” to “much better than expected outcome” (numbered –2 to +2). Follow-up times for participant evaluation are set after 3 months and 6 months. Goal attainment is evaluated after the defined time interval. At the end, the overall attainment score for all goals are calculated. In this study we will define up to 3 goals for PA [64]. The scale has been validated for use in rehabilitations populations [39] and has been used in studies with individuals with IDs [40,65].

##### Satisfaction With Life

This study will use the satisfaction with life scale developed by Bergström and Hochwälder [52], which was designed to assess satisfaction with the home environment and leisure time in individuals with mild to moderate IDs. The scale has 4 factors: (1) satisfaction with housing environment, (2) satisfaction with life, (3) satisfaction with meals, and (4) satisfaction with recreational activities. Items are read aloud by a researcher and answered by participants with 3 response options: good (happy face=2), in between (neutral face=1), or bad (sad face=0). In the current study, the scale is used to control for adverse effects.

#### Data Integrity

Patient- or proxy-reported and assessor-reported data are partly captured electronically using Research Electronic Data Capture (REDCap) (Vanderbilt University). REDCap is a web-based system that is compliant with relevant regulations and security requirements. The system has an integrated function for randomization. Questionnaires can be sent electronically to participants or the proxy respondent. Data not captured electronically, such as background information and physical performance test results, are registered at the baseline meeting. The study coordinator will evaluate the data of all participants for completeness. In cases of missing data, respondents will be contacted.

### Statistical Analyses

#### Sample Size

The study will be powered based on the primary outcome of accelerometer-measured steps per day (mean of 4 days) [4,66]. With a 2-group design and effect size of 0.8, power of 80%, and of .05, the expected minimum total sample size is 50 participants (25 participants in each group). The effect size in the current study is estimated based on previous studies, which have reported Cohen *d* values ranging from 0.29 to 0.91 [14,37,67]. An effect size of 0.8 is considered to be a clinically relevant difference between the 2 groups, corresponding to an increase in steps per day of approximately 2000 in the intervention group, which is also expected to be achievable [14]. To avoid underpowering the study and to prepare for expected dropout, we will recruit 30 participants per group, for a total sample size of 60 (Figure 3). The group size estimated is supported by other randomized controlled study protocols [27,37] and published results [68] of studies to enhance levels of physical activity in individuals with ID.

#### Data Analyses

The randomized controlled trial includes repeated measures in 2 groups, and linear mixed models will be used in the efficacy analyses of the intervention. In addition to a group variable (treatment or control), follow-up time (3 months and 6 months) and mean steps (with baseline comparison) will be added as covariates. An intention-to-treat approach will be used with a significance level of *P*<.05 and a secondary per-protocol analysis. All analyses will be performed using SPSS 26 software (IBM Corp).

## Results

The project is approved by the Regional Committee for Medical and Health Research Ethics in northern Norway and is registered at ClinicalTrials.gov. Enrollment was planned to start in April 2020 but will be delayed due to the pandemic situation. Participant recruitment for the randomized controlled study will be initiated as soon as practical difficulties due to the pandemic situation are solved. Participants will be recruited, randomized, and administered the intervention individually. The main contribution of this paper is a detailed plan to run our study, which will produce new knowledge about mHealth to support PA in individuals with IDs.

## Discussion

The present trial will investigate how modern technology and mHealth can be used in the promotion of PA in individuals with IDs. A tailored PA program is expected to increase levels of PA, and individuals with IDs and low PA have the greatest chances of improving [13]. Throughout our project, we have used an ecological approach, and we are currently developing a theory-based mHealth motivational support system for the promotion of PA, which we believe will increase the probability of improved levels of PA. Focusing on the communication abilities of each participant and individual goal setting may be particularly important [64]. As previous research has shown difficulties in recruitment and data collection, including missing follow-up data [13], we will be prepared to meet these challenges.

By including individuals with all types of IDs and low levels of PA, we can add to the knowledge on whether mHealth support can be successfully adjusted to individuals with different levels of functioning and whether it can increase levels of PA [5]. There is evidence demonstrating that an mHealth intervention for PA can improve self-efficacy in activities, social support [32], health conditions such as blood pressure [13], and the results of physical performance tests [69]. This study has potentially important implications for both individuals with IDs and their support networks. If successful, the project will provide a simple and accessible solution for promoting PA in individuals with IDs. For widespread clinical implementation, it is necessary to engage stakeholders who support individuals with IDs throughout their lives.

## Abbreviations

ABC: Aberrant Behavior Checklist
ABC-C: Aberrant Behavior Checklist—Community
ID: intellectual disability
mHealth: mobile health
PA: physical activity
SPPB: Short Physical Performance Battery
WHO: World Health Organization
